# Role of intensive care nurses on guiding patients’ families/relatives to organ donation

**DOI:** 10.12669/pjms.35.4.1285

**Published:** 2019

**Authors:** Ahmet Karaman, Neriman Akyolcu

**Affiliations:** 1 Ahmet Karaman, RN, MSc. Research Assistant, Surgical Nursing Department, Florence Nightingale Faculty of Nursing, Istanbul University-Cerrahpasa, Istanbul, Turkey; 2 Neriman Akyolcu, RN, MSc, PhD. Professor, Department of Nursing, Faculty of Health Sciences, Istinye University, Istanbul, Turkey

**Keywords:** Brain death, Intensive care nurse, Organ donation, Organ transplantation

## Abstract

**Objective:**

The aim was to determine the role of intensive care nurses on guiding the families/relatives of brain-death patients to organ donation.

**Methods:**

This research is a descriptive study. While the population of the study consisted of 1710 nurses working in the intensive care units of public, private and university hospitals in the city of Istanbul, the sample consisted of 353 intensive care nurses selected with stratified random sampling method from the probability sampling methods from this population. The data were collected by using “Data Collection Form”.

**Results:**

It was determined that 74.5% of the intensive care nurses carefully listened the family/relatives of the patient with possible brain death or suffering from brain death and supported them to express their emotion and thoughts clearly; when the family/relatives of the patients hospitalised in the intensive care unit wanted to get information about organ donation, 20.7% of the nurses made the preliminary explanation themselves and then guided the patient to an organ transplant coordinator for detailed information and 3.1% of the nurses generally gave this information themselves.

**Conclusions:**

It was determined that the knowledge of the intensive care nurses about brain death and organ donation was partially adequate and the function of guiding the families/relatives of brain-death patients to organ donation was mostly done by the physician.

## INTRODUCTION

Inadequacy of organ procurement from cadaver is a major problem throughout the world but this problem continues its existence in a much more serious dimension in developing countries such as Turkey and the efforts to solve this problem are ongoing.^1^,^2^

It is reported that the 81.1% of the organ donors are cadavers and 18.9% are living donors in European countries; 95% of the organ donors are cadevers in China; in Turkey on the other hand, 88.8% of the organ donors are living donors and 11.2% are cadavers. When these rates are compared, it is seen that while the rate of organ transplantation from living donor in Turkey has the highest statistics in Europe and China, cadaveric transplantation rate remains numerically far below from the donations in developed countries and thus this insufficiency appears as a much more important problem.^1^-^3^

In order to increase the rate of organ transplant from cadavers, it is very important to determine the patients who are terminally ill or have the possibility of brain death and prepare the patient’s family/relatives for this process as a result of diagnosing with brain death.^4^-^6^ In the intensive care units (ICUs) where healthcare professionals from different disciplines work and the brain death cases are most frequently encountered; in case of such a serious situation experienced by the family of the patients who are a potential organ donor with brain death or has the risk of brain death, the most important factor that will affect the family’s decision about organ donation is the attitude and persuasiveness of the ICU team and the feeling of trust they give.

ICU nurses can undertake a key role in the efforts of increasing organ donation since they are with the patient’s family/relatives for a long time and they are in a constant communication with them.^7^,^8^ The study was conducted to determine the role of ICU nurses in guiding the families/relatives of the brain-death patients to organ donation.

## METHODS

### Design and participants

This descriptive study was conducted in the hospitals located in European and Asian sides of Istanbul which is an economically rich cosmopolitan city with dense population located in the north-western Turkey. In the period of the study (29 December 2014- 10 March 2015), there were 386 hospitals (university, public, and private hospital) in Istanbul. In most of these hospitals especially in private hospitals, there were no ICUs or there were a few beds. Therefore, the population of the study consisted of 1710 nurses working in ICUs of 18 hospitals which have quality certificate and employing 30 or more ICU nurses. The size of the sample was calculated with *n* = *Nt*^2^*pq*/*d*^2^ (*N* − 1) + *t*^2^*pq* (*t*:1.96, *d*:0.05, *P* = 50%, *q* = 1 − *p*) formula and it was aimed to reach 326 ICU nurses. By considering the possibility of data loss, the study was completed with 353 ICU nurses (public hospital: 214; private hospital: 76 university hospital: 63).

The related trainings organized by their hospitals, the number of patients they provide care to in a shift, and the process hospitals follow in brain death cases are effective in order for ICU nurses to perform their roles in guiding the families/relatives of the brain-death patients to organ donation. In this context, these characteristics are different in public, university, and private sector. For this reason, the strata were created by considering the working status in the public, university, and private sectors. Weight of the stratum was 0.62 (public hospital), 0.21 (private hospital), 0.18 (university hospital). All hospitals planned for data collection except for one private hospital were reached. The working lists of nurses were obtained from the hospitals in the sample group and the nurses were selected from the strata using simple random sampling method.

### Data collection methods

The data were collected by conducting face-to-face interviews between 29 December 2014 and 10 March 2015 using a Data Collection Form prepared by the researchers in accordance with the literature.^9^-^11^

### Data collection tools

In the data collection form, there are questions about the descriptive characteristics, knowledge of ICU nurses about brain death and their practices in their units, responsibilities of ICU nurses about brain death and organ donation in their units.

### Data analysis

The data obtained from the study was analysed with the Statistical Package for Social Sience 21.0 packaged software. In the evaluation of the study data, the descriptive statistical methods, Kruskal Wallis test, Pearson’s Chi-square test, Yates Continuity Correction and Fisher’s Exact test were used. The results were evaluated at confidence interval of 95% and significance level of *p*<0.05.

### Ethical considerations

Written permission was obtained from clinical trials ethics committee (Ethical approval number: 83045809/604/01-01/118088) and all hospitals where the study data were collected. Before applying the data collection form, written and verbal consents of the ICU nurses were obtained.

## RESULTS

It was determined that 82.4% of the nurses formed the sample were 21-35 years old; 78.5% were female; 53% had a bachelor’s degree; 60% were working in public hospital; 39.7% had a professional experience of 1-5 years; 49.3% had the working experience of 1-5 years in ICU and one nurse provided care to an average of 3.082 ± 1.33636 patients in one shift.

It was found that 63.7% of the ICU nurses participating in the study did not have any training about brain death or organ donation. 77.4% of 128 nurses who stated that they received training on brain death or organ donation had that training in in-service training programs. It was determined that 40.8% of the nurses stated that their knowledge about brain death and organ donation was partially sufficient. 52.7% of ICU nurses participating in the study did not evaluate the patient in the units in terms of brain death.

It was seen that 80.2% of the ICU nurses stated that the opinions or approaches of the families/relatives of patients with possibility of brain death concerning organ donation were questioned. Seventy eight percent of the ICU nurses stated that the opinions or approaches of families/relatives of patients with possibility of brain death concerning organ donation were questioned in their unit expressed that this questioning was conducted by the physician; on the other hand, 4.2% said that this questioning was made by the nurse. It was determined that most nurses allowed family/relatives to see patients daily (67.1%), touch and communicate with patients (62%), informed about patient’s condition (47%), explained treatment and care interventions (42.8%). It was found that most nurses listened and provided support (74.5%). However, for those who don’t, half of them thought it was not their responsibility (54.4%); most nurses did not stay with family when brain death explained (74.5%) and did not think it is their responsibility (77.6%) (Table-I). The level of education of nurses has no connection with care interventions (p>0.05) as shown in Table-II. As the duration of professional experience increases, the rate of evaluation of nurses in terms of brain death has increased (Table-II).

**Table I:** The distribution of care interventions of the ICU nurses for the families/relatives of patients with brain death or possible brain death (n=353).

Care Interventions	n	%
*What do you do to make patient’s family/relatives feel that you are providing qualified care to the patient? (n=353)*		
I allow the family/relatives to see the patient every day	237	67.1
I allow the family/relatives to touch and communicate with the patient	219	62
I inform the patient’s family/relatives every day about the patient’s condition	166	47
I explain the treatment and care interventions to the patient even if he/she is unconscious.	151	42.8
I provide the involvement of patient’s family/relatives into the patient’s care	140	39.7
*More than one response was given		
*Do you listen the patient’s family/relatives carefully and support them to express their emotions and thoughts ? (n=353)*		
Yes	263	74.5
No	90	25.5
Total	353	100
*If your answer is no, explain the reasons (n=90)*		
I do not think I have such a responsibility	49	54.4
I do not have time for families/relatives of patients because I have too many responsibilities for patient care	33	36.7
Since the patient’s condition is severe, I refrain from talking to the patient’s family/relatives thinking that they will be upset	14	15.6
I am having difficulty in drawing the line when communicating with the family/relatives of patient with brain death or possible brain death	10	11.1
Due to the special condition of such patients, communicating with the patient’s family/relatives make me very emotionally deprived	5	5.6
*More than one response was given		
*Do you stay with the patient’s family/relatives when the diagnosis of brain death is explained to them?(n=353)*		
Yes	90	25.5
No	263	74.5
*If your answer is no, explain the reasons (n=263)*		
I do not think it’s the responsibility of the nurse.	204	77.6
It is the responsibility of the nurse, but I can not allocate time because of the workload	47	17.9
I am disturbed to see the reactions of the patient and his/her family at that time	27	10.2
*More than one response was given		
*What do you do when the patient’s family/relatives want to get information about organ donation? (n=353)*		
I guide them directly to the ICU physician	112	31.4
I make the preliminary explanation myself and guide them to the organ transplant coordinator for detailed information	73	20.7
I guide them to the organ transplant coordinator directly.	116	32.9
I make the preliminary explanation myself and guide them to the ICU physician for detailed information	38	10.8
I usually give this information myself	11	3.1
I do not response their request due to workload	3	0.8

**Table II:** Comparison of educational level, professional experience, and the status of receiving training on brain death and organ transplant with the applied care interventions

Care Interventions	Educational level							

	Medical Vocational High School / College	Associate Degree	Bachelor’s degree	Postgraduate				

	n (%)	n (%)	n (%)	n (%)				p
*Do you evaluate the patient in terms of brain death?*								
Yes	40 (54.1)	20 (45.5)	80 (42.8)	27 (56.3)				^b^0.213
No	34 (45.9)	24 (54.5)	107 (57.2)	21 (43.8)				
*Do you listen patient’s family/relatives carefully and support them to express their emotions and thoughts?*								
Yes	52 (70.3)	32 (72.7)	140 (74.9)	39 (81.3)				^b^0.586
No	22 (29.7)	12 (27.3)	47 (25.1)	9 (18.8)				
*Do you stay with the family/relatives when the physician explain the diagnosis of brain death to them?*								
Yes	24 (32.4)	9 (20.5)	43 (23.0)	14 (29.2)				^b^0.331
No	50 (67.6)	35 (79.5)	144 (77.0)	34 (70.8)				
*What do you do when patient’s family/relatives want to get information about organ donation?*								
I guide them to the ICU physician directly.	27 (36.5)	13 (29.5)	61 (32.6)	11 (22.9)				0.447
I make the preliminary explanation myself and guide them to the organ transplant coordinator for detailed information.	15 (20.3)	4 (9.1)	38 (20.3)	16 (33.3)				0.04*
I guide them to the organ transplant coordinator directly .	23 (31.1)	16 (36.4)	62 (33.2)	15 (31.3)				0.937
I make the preliminary explanation myself and guide them to the ICU physician for detailed information .	7 (9.5)	8 (18.2)	20 (10.7)	3 (6.3)				0.299
I usually give this information myself.	2 (2.7)	3 (6.8)	4 (2.1)	2 (4.2)				^d^0.310
I do not response their request due to the intensive workload.	0	0	2 (1.1)	1 (2.1)				^d^0.677

Care Interventions	Years of professional experience				Receiving training on brain death and organ donation		p

	Less than 1 year	Between 1-5 years	Between 6-10 years	11 years and more	Yes	No	

	n (%)	n (%)	n (%)	n (%)	p	n (%)	n (%)	
*Do you evaluate the patient in terms of brain death ?*								
Yes	7 (23.3)	68 (48.6)	63 (51.6)	29 (47.5)	^b^0.048*	68 (53.1)	99 (44.0)	^b^0.099
No	23 (76.7)	72 (51.4)	59 (48.4)	32 (52.5)		60 (46.9)	126 (56.0)	
*Do you listen patient’s family/relatives carefully and support them to express their emotions and thoughts?*								
Yes	19 (63.3)	103 (73.6)	93 (76.2)	48 (78.7)	^b^0.425	105 (82.0)	158 (70.2)	^e^0.014*
No	11 (36.7)	37 (26.4)	29 (23.8)	13 (21.3)		23 (18.0)	67 (29.8)	
*Do you stay with the family/relatives when the physician explain the diagnosis of brain death to them?*								
Yes	5 (16.7)	28 (20.0)	41 (33.6)	16 (26.2)	^b^0.053	44 (34.4)	46 (20.4)	^b^0.004**
No	25 (83.3)	112 (80.0)	81 (66.4)	45 (73.8)		84 (65.6)	179 (79.6)	
*What do you do when patient’s family/relatives want to get information about organ donation?*								
I guide them to the ICU physician directly.	11 (36.7)	50 (35.7)	36 (29.5)	15 (24.6)	^b^0.380	37 (28.9)	75 (33.3)	^b^0.39
I make the preliminary explanation myself and guide them to the organ transplant coordinator for detailed information.	7 (23.3)	24 (17.1)	31 (25.4)	11 (18.0)	^b^0.373	31 (24.2)	42 (18.7)	^b^0.216
I guide them to the organ transplant coordinator directly.	5 (16.7)	48 (34.3)	34 (27.9)	29 (47.5)	^b^0.012*	49 (38.3)	67 (29.9)	^b^0.102
I make the preliminary explanation myself and guide them to the ICU physician for detailed information .	4 (13.3)	13 (9.3)	16 (13.1)	5 (8.3)	^b^0.649	9 (7.0)	29 (12.9)	^e^0.126
I usually give this information myself.	3 (10.0)	4 (2.9)	3 (2.5)	1 (1.6)	^d^0.183	2(1.6)	9 (4.0)	^d^0.34
I do not response their request due to the intensive workload.	0	1 (0.7)	2 (1.6)	0	^d^0.691	0	3 (1.3)	^d^0.556

b Pearson Chi-Square Test,

d Fisher-Freeman-Halton test, d Fisher’s Exact Test,

e Yates’s Continuity Correction Test,

* p<0.05

** p<0.01

## DISCUSSION

It was determined in this study that majority of the ICU nurses participating in the study received no training on brain death or organ donation. The result which is different from the results of the studies^12^-^14^ determining that most of them get information is partially similar to the results of the study by Yilmaz E.^15^ The results indicating that they received no training on brain death or organ donation and the knowledge of a great majority of those who received this training was inadequate and partially sufficient can be evaluated as an unexpected and ignored situation when considering the problems in the organ donation and the positional characteristic of the population of the study across Turkey.

In the study by Collins TJ^16^, it was also observed that 55% of ICU nurses did not follow up patients in terms of brain death. It was determined in this study that little more than half of the ICU nurses participating in the study did not evaluate the patient in terms of brain death in their units. The result indicating that the rate of nurses, who were keeping the patient under 24-hour supervision in the ICU team, evaluating the patient in terms of brain death was low thus emphasizing the important and necessity to increase awareness of the nurses about evaluating the patient in terms of brain death in order to determine the potential organ/tissue donors early, to start the organ donation process as soon as possible, and to provide positive results.

It was determined in this study that close to all ICU nurses expressed that the opinions or approaches of the family/relatives of patient, having the possibility of brain death, about organ donation were questioned in their units; on the other hand, majority of the nurses saying that the questioning was made stated that this process was made by the physician. The result stating the rate of questioning the family/relatives for tissue/organ donation by the nurses, who have the most communication with the patient and his/her family/relatives and have the opportunity to diagnose them in all aspects, was low can be interpreted as a concrete indication that nurses did not take responsibility about this issue or could not take it due to hard working conditions.

In the study by Taylor P, Young K, & Kneteman N^17^, it was observed that the family/relatives of the brain-death patient gave consent to tissue/organ donation at a higher rate in the ICUs where a better communication was established with the relatives of the donor candidates. In the study by McCoy and Bell (1994) cited in the study by Unal S, Elyas Z, Kaya Y, & Ozcan C^18^, entitled “beliefs and attitudes of healthcare professionals about brain death and organ donation”, it was determined that as the interaction levels of nurses with organ donors or recipients increased, their attitudes towards organ donation became more positive. In this study, it was determined that in order for the ICU nurses to make the family/relatives of patient with brain death or possible brain death feel in this critical period that the patient was provided a safe, efficient, and qualified care; more than half of the ICU nurses allowed the family/relatives to see the patient every day, to touch and communicate with the patient, listened the patient’s family/relatives carefully in this process and supported them to express their emotions and thoughts clearly, close to half of the nurses informed the patient’s family/relatives about the condition of the patient every day and allowed the involvement of the patient’s family/relatives into the patient’s care. The study result showing that the nurses partially fulfilled the care interventions required to be applied to the brain-death patients and their families/relatives but not at the desired level made us think that the nurses did not give sufficient importance to this subject or they ignored it due to intensity of the working conditions.

It was also observed that over half of the ICU nurses were not with the family/relatives while the physician was explaining the diagnosis of brain death. The result of the study was found to be important in terms of the ICU nurses’ seeing the reactions of the individual’s family/relatives towards the diagnosis of brain death and demonstrating approaches according to these reactions as well as providing the family/relatives to trust the nurses and revealing the inadequacies.

The ICU nurses who had more professional experience duration had more ratios evaluating the patients in terms of brain death. The results obtained concerning the comparison of the professional experience duration of the nurses with their care interventions applied to the families/relatives of the patients with brain death or the possibility of brain death can be evaluated as a concrete result showing the effectiveness of experience even if it was not in all care interventions.

The status of making the interventions “listening patient’s family/relatives carefully and supporting them to express their emotions and thoughts” and “being with the family/relatives of the patient while the physician explains the diagnosis of brain death to them” was determined to be significantly higher in the nurses expressing that they did not receive the training after graduation compared to those who received the training (*p*<0.05). When considering that both care initiatives are the accurate approaches and those who do not receive the training after graduation constituted the majority, it can be asserted that postgraduate training programs should be updated, developed and repeated.

## CONCLUSIONS

In conclusion, it was determined that the knowledge of the intensive care nurses about brain death and organ donation was partially adequate and the function of guiding the families/relatives of brain-death patients to organ donation was mostly done by the physician.

In accordance with all these results; It can be recommended to

- Provide comprehensive trainings to ICU nurses including the process of brain death diagnosis and tissue/organ donation through in-service trainings after graduation,
- Organise in-service training, conference, course, etc., aiming for ICU nurses to gain communication skills necessary to establish an effective communication with the family and relatives of the patient with brain death or the possibility of brain death, and to organize comprehensive, instructive, informative and thought-provoking symposiums to raise awareness on fulfilling role model functions regarding tissue/organ donation and transplantation,
- Establish the institutional protocols in health institutions about this issue by determining the responsibilities of all ICU team in the process followed in the care of patients with brain death or the possibility of brain death and their families/relatives in ICUs.

## References

[1] Republic of Turkey Ministry of Health Turkey Organ and Tissue Information System.

[2] Eurotransplant Transplants, deceased donors from all ET, by year, by organ Combination.

[3] Ahmad HR, Haider SG, Rizvi A, Naqvi A (2019). When is deceased organ donation suitable?A critical reflection on medical criteria. Pak J Med Sci.

[4] Bharambe VK, Arole VU, Puranam V, Kulkarni PP, Kulkarni PS (2018). Knowledge and attitude toward organ donation among health-care professionals in a rural town in India. SJKDT.

[5] Floden A, Berg M, Forsberg A (2011). ICU nurses'perceptions of responsibilities and organisation in relation to organ donation—A phenomenographic study. ICCN.

[6] Oluyombo R, Fawale MB, Ojewola RW, Busari OA, Ogunmola OJ, Olanrewaju TO (2016). Knowledge regarding organ donation and willingness to donate among health workers in South-West Nigeria. IJOTM.

[7] Camut S, Baumann A, Dubois V, Ducrocq X, Audibert G (2016). Non-therapeutic intensive care for organ donation:A healthcare professionals'opinion survey. Nurs Ethics.

[8] Collins T (2012). Strategies to increase organ donation:The role of critical care practitioners. Nurs Critical Care.

[9] Dominguez-Gil B, Murphy P, Procaccio F (2016). Ten changes that could improve organ donation in the intensive care unit. ICM.

[10] Meyer K, Bjork IT, Eide H (2012). Intensive care nurses' perceptions of their professional competence in the organ donor process: A national survey. JAN.

[11] Tamburri LM (2006). The role of critical care nurses in the organ donation breakthrough collaborative. CCN.

[12] Demir T, Selimen D, Yildırım M, Kucuk HF (2011). Knowledge and attitudes toward organ/tissue donation and transplantation among health care professionals working in organ transplantation or dialysis units. Transplant Proc.

[13] Kocak A, Aktas EO, Senol E, Kaya A, Bilgin UE (2010). Ege University Faculty of Medicine undergraduates'knowledge level regarding organ donation and transplantation. Ege J Med.

[14] Sapulu Y (2011). Knowledge, attitudes and approaches of intensive care nurses in organ and tissue transplantation and donation [unpublished master thesis].

[15] Yilmaz E (2006). Thoughts of health workers on organ transplantation and donation [unpublished master thesis].

[16] Collins TJ (2005). Organ and tissue donation:A survey of nurse's knowledge and educational needs in an adult ITU. ICCN.

[17] Taylor P, Young K, Kneteman N (1997). Intensive care nurses'participation in organ procurement:Impact on organ donation rates. Transplant Proc.

[18] Unal S, Elyas Z, Kaya Y, Ozcan C (2010). Beliefs and attitudes of healthcare personnel regarding brain death and organ donation. F. U. J Soc Sci.

